# Neurobiological Basis of Language Learning Difficulties

**DOI:** 10.1016/j.tics.2016.06.012

**Published:** 2016-09

**Authors:** Saloni Krishnan, Kate E. Watkins, Dorothy V.M. Bishop

**Affiliations:** 1 Department of Experimental Psychology, University of Oxford, 9 South Parks Road, Oxford OX1 3UD, UK

**Keywords:** specific language impairment, dyslexia, development, striatum, disorder, subcortical, procedural learning

## Abstract

In this paper we highlight why there is a need to examine subcortical learning systems in children with language impairment and dyslexia, rather than focusing solely on cortical areas relevant for language. First, behavioural studies find that children with these neurodevelopmental disorders perform less well than peers on procedural learning tasks that depend on corticostriatal learning circuits. Second, fMRI studies in neurotypical adults implicate corticostriatal and hippocampal systems in language learning. Finally, structural and functional abnormalities are seen in the striatum in children with language disorders. Studying corticostriatal networks in developmental language disorders could offer us insights into their neurobiological basis and elucidate possible modes of compensation for intervention.

## Trends

Individuals with SLI and dyslexia have impaired or immature learning mechanisms; this hampers their extraction of structure in complex learning environments.

These learning difficulties are not general or confined to language. Problems are specific to tasks that involve implicitly learning sequential structure or complex cue–outcome relationships. Such learning is thought to depend upon corticostriatal circuits.

In language learning studies, the striatum is recruited when adults extract sequential information from auditory-verbal sequences and as they learn complex motor routines relevant for speech.

Neuroimaging studies indicate striatal abnormalities in individuals with language disorders.

There is a need to probe the integrity of neural learning systems in developmental language disorders using tasks relevant for language learning which place specific demands on the striatum/MTL.

Specific language impairment (SLI) and developmental dyslexia are categorized as types of specific learning disability (Box 1), but for many years the bulk of research on these disorders has looked at perceptual impairments and problems with specific linguistic components such as phonology and grammar. Here we adopt a different perspective: the idea that children with these disorders have impairments in the basic process of learning language. On this view, the language and literacy difficulties experienced by these children are not byproducts of some other primary deficit; instead, they implicate impairment or immaturity of learning mechanisms that allow the extraction of structure from a rich and varying language environment. We review recent developmental and neurobiological studies to evaluate the contribution of different brain systems in language learning, particularly focusing on how they might be affected in children with language and reading disorders.

## What Learning Impairments Are Observed in Developmental Language and Reading Disorders?

Children with developmental language disorders struggle to learn new words [1] and syntactic constructions [2]. Is this a linguistic problem, or do they exhibit difficulties with learning new information more generally? Learning is not a unitary phenomenon. Neuropsychological studies have suggested functional and neurological distinctions between different types of learning (Box 2). Ullman and Pierpont [3] were the first to suggest that the procedural learning system, which is involved in implicit learning, was impaired in individuals with SLI. They proposed that procedural impairments could account for poor learning of grammatical rules, such as the past tense inflection of regular verbs (also see [4]). The postulated impairments in procedural learning were not, however, specific to language; they would have broader effects, with deficits predicted in the acquisition of any skill involving sequences – irrespective of whether the sequences were sensorimotor or abstract. By contrast, declarative learning systems, which support the sort of idiosyncratic mapping required to learn new vocabulary or inflection of irregular verb forms, were argued to be relatively intact.

The procedural deficit hypothesis inspired a series of studies examining the non-linguistic procedural learning abilities of children with SLI, typically using a **serial reaction time** (SRT) paradigm (see Glossary). A meta-analysis of eight studies using an SRT paradigm with children with SLI and age-matched controls revealed small but significant effects of language impairment on this task [5], of the order of 0.33 of a standard deviation. In studies with younger participants, larger effect sizes were found. In SLI, learning was impaired more when sequences were long and complex [6]. Similar problems with learning implicit sequences in the SRT task are also seen in younger typically developing children matched on grammatical ability, suggesting that implicit sequence learning in SLI may be immature rather following an atypical developmental trajectory [7].

The learning abilities of individuals with dyslexia have also been examined using SRT measures, motivated by broader theories suggesting that the automatisation of learning is impaired in this disorder [8]. A meta-analysis of nine studies that used SRT paradigms with individuals with dyslexia revealed a moderate effect of having dyslexia (0.45 of a standard deviation) [9]. This meta-analysis also indicated that age and sequence type influenced the likelihood of finding a difference between dyslexic and control groups.

SRT paradigms emphasise the motor aspects of procedural learning. However, these groups also show learning impairments in non-motor paradigms. Both children with dyslexia [10] and adults with SLI 11, 12 appear to have difficulty extracting structure from novel sequences in **artificial grammar learning** (AGL) paradigms. These difficulties in making judgements about grammaticality are not related to problems holding information in mind because deficits are present even when children with dyslexia can accurately recall a training sequence from memory 10, 13. Other studies have found that children with SLI perform worse than typically developing peers at extracting regularities from speech streams in **statistical learning** paradigms [14], although they can extract relevant information when exposure is doubled [15]. Adults with dyslexia also find extracting regularities in these statistical learning tests difficult, and their performance correlates with their reading ability [16]. Although these tasks stretch the definition of procedural learning provided by Ullman [3], there is some indication that the same implicit learning processes are involved (Box 3).

**Figure 1:**
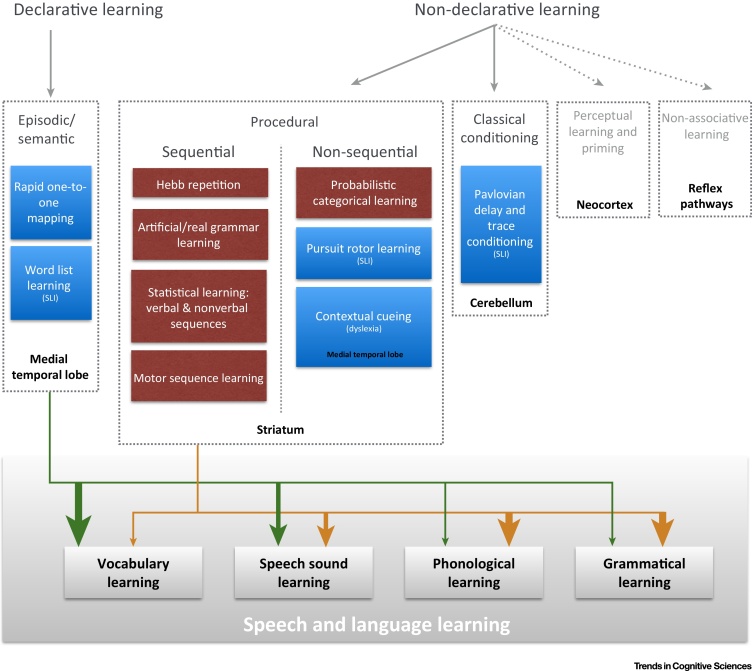
Contribution of Learning and Memory Systems to Language Learning Difficulties. The top panel of the figure shows different aspects of declarative and non-declarative learning systems [102]. Boxes indicate the types of tasks that have been used to assess each of these forms of learning in children with language and reading disorders (some aspects of non-declarative learning were beyond the scope of this review, these are indicated by dashed arrows). Task boxes are coloured in blue if no learning impairment (with reference to learning rates, rather than overall performance) was observed when controlling for age, IQ, and working memory in children with language or reading disorders, and coloured in red if children with specific language impairment (SLI) or dyslexia did not learn as well as their typically developing peers. Children with language learning difficulties perform poorly on procedural learning tasks, particularly those that are sequential or involve complex categorical learning. The brain structures thought to be especially important for each form of learning are indicated in black text (as shown in [102]). However, these regions are not isolated during nor solely responsive for such learning; instead, they should be considered as key hubs within an interconnected learning system. The bottom panel shows the hypothesised contribution of these learning systems to different aspects of language learning, emphasising the interactions between declarative (green lines) and procedural learning systems (orange lines). The weight of the arrows represents the potential strength of the contribution, with thicker arrows denoting greater contribution – these are illustrative and drawn on the basis of studies reviewed in Neurobiological Systems Involved in Language Learning.

The aforementioned implicit learning studies have shown that those with language disorders are less able to learn regularities in sequences, even when these are non-linguistic (summarised in Figure 1). Difficulties with sequential learning are not confined to the encoding stage. There is emerging evidence that individuals with SLI 17, 18 and dyslexia [19] do not consolidate and retain sequence knowledge as effectively as other children. There is some evidence for these learning deficits patterning with individual differences in grammatical skill 20, 21, 22, but not vocabulary 20, 23, in children with SLI (although for robust evidence of such associations we will need to develop psychometrically strong indices of procedural learning in individuals).

## Specificity of Learning Difficulties in Developmental Language Disorders

Although the literature reviewed above suggests that children with SLI and dyslexia are impaired in sequential procedural learning tasks, these deficits could simply indicate a generalised learning deficit. In the following we argue that this is not the case, on the basis of studies that have probed declarative learning as well as non-sequential procedural learning.

Declarative learning is thought to be an area of relative strength in children with SLI and dyslexia 3, 24. Despite this, relatively few studies have empirically examined declarative learning in these groups. In tasks that involve encoding and retrieving word lists, children with SLI perform poorly relative to age-matched controls 22, 25, 26. However, individual differences in working memory seem to account for these differences 22, 25, 26, suggesting that the ability to hold information in mind for short periods of time may be the limiting factor for declarative learning. Another study demonstrated that children with SLI and controls show equivalent non-linguistic **paired associate learning** [26]. In addition, this study showed that their rate of learning verbal–visual mappings over four sessions is comparable to their typically developing peers, although their initial learning of these mappings was more severely affected [26]. Children with dyslexia also show equivalent learning to age-matched peers on visual-visual paired-associates learning, but less well when verbal-visual or verbal-verbal mappings must be made [27]. These results suggest that paired-associated learning is impaired when it requires learning of a novel sequence of speech sounds – which taxes the procedural system – but that learning of arbitrary associations, which employs declarative learning, is intact. Further evidence that declarative memory is unimpaired comes from a study reporting that, when an implicit AGL task is made explicit, learning differences are no longer seen in a group of adults with dyslexia [28].

In addition to relative strengths in declarative memory, not all forms of implicit or procedural learning are impaired in individuals with developmental language disorders (Figure 1). Both children and adults with dyslexia showed similar implicit learning to controls in non-sequential **contextual cueing** tasks 29, 30, 31. Children with SLI also show learning similar to that of age-matched controls in other non-sequential procedural learning tasks such as the **pursuit rotor task** ([7], but see [32]); they do not differ from controls in **eyeblink conditioning**, which engages corticocerebellar circuits 33, 34. However, a sequential learning deficit cannot explain all the evidence. **Probabilistic category learning tasks**, such as the ‘weather prediction’ task, have also been used to probe procedural learning in these groups. Adults with dyslexia 35, 36 or SLI [37], but see [38] do not acquire implicit categorical knowledge at the same rate as age-matched controls. One possibility is that individuals with language disorders struggle in conditions where learning dimensions are not explicitly defined. Another is that these learning deficits occur concurrently with core sequence learning difficulties, perhaps owing to impairment in overlapping neural circuits (Box 3).

In summary, individuals with language and literacy disorders have difficulties with procedural learning in sequence-based tasks, but appear to be relatively unimpaired on declarative and non-sequential procedural learning measures. This may explain why their difficulties are more prominent in language tasks – which heavily load on to extracting and producing sequential information. However, no type of learning is purely declarative or procedural in nature, and the ways in which these distinctions apply to language learning in particular needs clarification.

## Neurobiological Systems Involved in Language Learning

**Figure 2:**
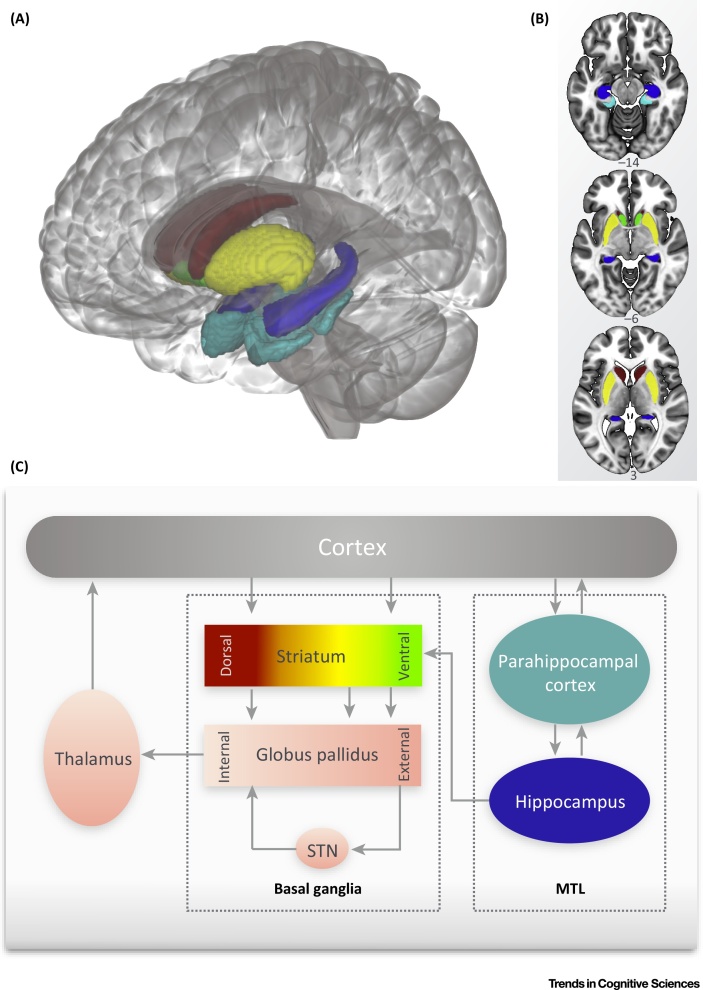
Corticostriatal and Hippocampal Learning Systems and Connections. (A) 3D representation of the striatum and medial temporal lobe (MTL) shown within a glass brain. The coloured areas on the image correspond to the labels in the schematic below. Blue, hippocampus; cyan, parahippocampal gyrus (anterior and posterior regions); green, nucleus accumbens which is part of the ventral striatum; red, caudate nucleus; yellow, putamen (the dorsal striatum includes the caudate nucleus and the putamen). (B) The striatum and MTL shown on 2D axial slices. Colours correspond to those in the 3D representation. (C) Schematic representation of the connections between the cortex, basal ganglia, and the MTL. The nuclei not shown in the 3D representation are coloured pink.

Language learning involves many different processes, such as extracting implicit knowledge about how sequences of sounds and words combine, learning novel mappings between words and referents, and consolidating learned knowledge to make it readily accessible (Box 4). We review here how neurobiological learning systems are involved in some of these different aspects of language learning, and how these map onto conventional knowledge about the roles of these systems (as outlined in Box 2, Box 3). Although we describe differences in the structure and function of the striatum and hippocampus, these structures are connected to each other as well as to the cortex and other subcortical structures (Figure 2). Functional interactions between these regions have been described during learning [39]. Consequently, changes in functional neural activity in one of these regions during language learning do not imply that this region is solely responsible for that type of learning, but rather that this might reflect a local change within a hub of a broader learning network.

The extraction and encoding of verbal sequential regularities is particularly relevant to learning the phonology and grammar of a language. These are learned implicitly, and can be considered as examples of procedural learning. The frontal cortex and the basal ganglia appear to be relevant to such learning 40, 41, 42. For example, the left inferior frontal gyrus and the bilateral striatum are recruited for statistical learning of word boundaries in an artificial language [42]. People with striatal degeneration are impaired at using sequential regularities in artificial speech streams to derive ‘morpho-syntactic’ rules and ‘words’ [43]. However, extracting sequential regularities is not purely dependent on the striatum, and declarative memory systems also show some involvement in this process. A study using a **Hebb repetition learning** task replicated findings of a correlation between striatal activation and learning [44]. Nevertheless, multivariate analyses revealed that the hippocampus and medial temporal lobe (MTL) were coding the identity of repeating sequences. More recently, overlapping spatiotemporal networks that include auditory cortex, regions considered to be part of the dorsal speech (or auditory)-processing stream [45], including the striatum and the hippocampus, have been shown to be differentially engaged as people learn to identify ‘words’ in an artificial language [46]. Similar cortical results have been shown using a natural language task [47] and artificial grammar learning paradigms [48]. These findings indicate that interactions between corticostriatal and corticohippocampal regions occur over the course of learning.

Word learning involves mapping a novel sequence of sounds to a referent. Learning arbitrary mappings is a classic ‘declarative’ task, and there is ample evidence suggesting that the hippocampus is an important region for encoding such mappings. For example, in an fMRI study examining how adults learn new vocabulary, activity over the left hippocampus and fusiform gyrus declined as associations between pseudowords and pictures were repeated [49]. Other word learning studies have shown that hippocampal activity at the encoding stage relates to whether words are subsequently recalled [50] or recognised [51]. Davis and Gaskell [52] have suggested a two-stage account for word learning, where rapid initial learning dependent on the hippocampus is followed by a slower consolidation process where there is a transfer of learnt information to the cortex, particularly superior temporal, inferior frontal and premotor regions.

It would be rash to conclude that the hippocampus is necessary and sufficient for word learning. Studies on patient H.M. (Box 2) indicate that residual semantic learning is present, despite his bilateral and complete hippocampal lesions [53]. In addition, cases with bilateral hippocampal damage sustained in childhood perform at average to low-average levels on standardised verbal measures [54], suggesting that semantic learning can rely upon areas adjacent to the hippocampus within the MTL.

Furthermore, regions involved in word learning extend beyond the MTL. Recent work shows that creating sound–meaning links also recruits the striatum. The ventral striatum (nucleus accumbens) is activated as these links are learned, suggesting a role for reward-based circuitry in learning novel words [55]. The dorsal striatum also responds to feedback in verbal paired-associate tasks [56], especially when participants believed that the feedback was indicative of achievement [57]. The striatum is also recruited when learning to produce novel words. Activity in the striatum decreases as people covertly repeat words in their native language [58], as well as when they learn words in a non-native language [59]: this reflects articulatory learning of the sequence of sounds, from an initial phase of sequencing novelty to habitual performance of an utterance. This is evidence that both corticostriatal and corticohippocampal networks are involved in word learning, although they seem to be tied to different aspects of this process. Corticostriatal networks are responsive to the motor and sequential demands of word learning, with some indication that reward-related circuitry might play a role in sound–meaning mapping.

When learning a language, listeners must also learn to group sounds they hear into the categories relevant in that language (see speech sound learning, Box 4). Given that speech sounds are multi-featured and variable, single acoustic features cannot be used to learn these distinctions. Learning occurs in a probabilistic fashion and theoretically should involve procedural learning systems. A few studies have explored the brain systems involved in speech category learning 60, 61. A recent study examining the dynamics of non-native speech category learning in adults [60] showed that this learning is initially associated with activation in both hippocampal and corticostriatal circuits. Across learning trials, participants’ behavioural responses indicated a shift from a rule-based strategy to one that is more procedural. In line with the crossover to a procedural strategy, the corticostriatal system showed increased activation during learning and was associated with better categorisation performance.

In summary, domain-general learning mechanisms involving striatal and MTL circuits are also recruited for speech and language learning. Corticostriatal systems are involved when adults learn speech sequences for articulation and when complex regularities in auditory sequences must be extracted. MTL circuits are relevant for learning arbitrary and explicit however, no single speech or language behaviour is associated with corticostriatal or MTL circuits alone; instead there are interactions between these learning systems as language is learned.

## Subcortical Abnormalities in Individuals with Language Disorders

Given the difficulties in language learning experienced by children with SLI, we might expect them to exhibit structural or functional differences in neurobiological learning circuits (Figure 2 depicts the connections between the MTL, striatum, and the cortex). A simple prediction based on their behavioural profile is that they should show abnormalities in the basal ganglia, but their hippocampi and medial temporal cortices will resemble those of age-matched controls. However, given that SLI and dyslexia are neurodevelopmental language disorders, we might expect the profiles of impairments to change during development.

The majority of studies on the brain bases of SLI and dyslexia focus on cortical anatomy, with particular reference to hemispheric asymmetries 62, 63. However, the neurobiological literature needs to be interpreted cautiously, given the inconsistencies in the direction of results, the small numbers in each group, the heterogeneity in defining the disorder, and the different age-ranges used across different studies. Bearing in mind these caveats, there is evidence of subcortical abnormalities or atypicalities in individuals with SLI, particularly in the striatum (Box 2 for the role of the basal ganglia in learning). Studies converge to indicate that the volume of the caudate nucleus is altered in children with SLI relative to typically developing peers 64, 65, 66, 67. Some studies suggest that a reduction in volume is observed 64, 65, which would pattern with the bilateral reductions in caudate nucleus volumes observed in affected members of the KE family (who have severe speech and language problems in the context of a rare genetic mutation) [68]. However, others have reported increases in caudate nucleus volume 66, 67. The changes in directionality of differences might be accounted for by differences within the analysis pipeline across these studies. The available literature also indicates that striatal differences are affected by age. Early differences observed in striatal volumes between children with SLI and typically developing children appear to normalise by late adolescence 66, 69, although longitudinal studies are necessary to confirm this point.

In contrast to the findings from individuals with SLI, structural differences in the striatum [70] are only inconsistently observed in those with dyslexia. A recent well-powered cross-linguistic study found only one regional difference – reduced grey matter in the left thalamus [71]. With respect to language processing, stimulation studies of the thalamus indicate a ‘specific alerting response’, which could gate the entry of language information to the perisylvian cortex, and is implemented via thalamic connections to the striatum and cortex [72]. The alerting response is thought to accelerate language and memory processes because gating of different cortical networks could allow enhanced encoding and retrieval of specific memories [72]. However, even this structural difference is not observed across all studies. A possible explanation for lack of consistent structural differences is the behavioural heterogeneity displayed by this group [73]. While phonological skills are thought to be impaired in those with dyslexia, dyslexic readers do not struggle with identical aspects of phonology [74] and there are children with reading disorders who have unimpaired phonology [75]. In addition, dyslexia results from a combination of multiple risk factors, including phonological problems, as well as motor, oral language, and executive functioning deficits [76]. Studies comparing dyslexics to controls may therefore be grouping together individuals with varying aetiologies.

Functional studies, however, have indicated that adults with dyslexia show hyperactivation of the striatum [63]. This striatal overactivity was not seen in children with dyslexia, leading the authors to suggest that striatal overactivity may be a compensatory mechanism in adulthood. In line with this, a recent study suggests that children with dyslexia show striatal overactivity when phonological tasks are simple but not when they are complex [77]. Functional studies of children with SLI also report increased activity in the head of the right caudate nucleus for phonological [78] and executive tasks [79].

Striatal changes may not suffice to cause language disorders. In the study by Badcock and colleagues [65], the unaffected siblings of children with SLI also had significant reductions in the volume of the caudate nucleus relative to typically developing children. It is possible that striatal abnormalities act as a heritable risk factor for language disorders, but other risk factors are necessary before the disorder manifests. If this is the case, some neurological differences may be protective. A recent structural network analysis showed that the hippocampus, temporal pole, and putamen were less strongly connected in individuals with a higher risk for dyslexia relative to those with low risk [80]. Intervention studies with dyslexia suggest that hippocampal volumes are enlarged after training where behavioural gains are made, suggesting successful compensatory change 81, 82. Finally, structural and functional differences between children with SLI and controls have been reported in inferior frontal [83], temporal 78, 84, and inferior parietal cortex 85, 86, and in white matter tracts connecting these regions 87, 88. These differences suggest that it is important to consider the entire learning system, including regions of the brain that might be involved in the consolidation and storage of linguistic and sequential knowledge.

A cautionary note from this area of research is that findings from brain imaging do not consistently replicate [62]. There is a need for well-powered neuroimaging studies to address brain–behaviour relationships in language disorders, allowing us to take into account the heterogeneity of language disorders and their diagnosis. To ensure that fMRI findings are not simply descriptive of a specific sample, we need to test whether fMRI findings generalise beyond the tested sample, scanner, and stimuli used (see [89] for related recommendations).

## Concluding Remarks and Future Directions

Individuals with SLI and dyslexia have difficulties in performing sequential procedural tasks and learning from feedback, but not in simple mapping tasks or non-sequential implicit learning. In language learning tasks, corticostriatal systems have been shown to be involved in acquiring complex motor routines that are relevant to speech and in learning speech categories from feedback. Given the evidence of abnormalities in the structure and function of corticostriatal systems in developmental language disorders, a plausible bridging hypothesis is that dysfunctions of corticostriatal systems can explain difficulties in learning language. These difficulties are likely to have greater impact on aspects of language that involve learning complex rules that are probabilistic and sequential, such as phonotactics and morpho-syntax, but would also affect the ease with which learned motor skills become habitual.

A facet that is currently missing from the literature is that both neurobiological and behavioural studies in these groups suggest that the influence of corticostriatal learning systems, and their impact on behaviour, changes substantially with age. There is a need for longitudinal studies in this area – to explore the trajectory of corticostriatal dysfunctions during development as well as how these pattern with learning behaviour. Such studies would also be helpful in establishing whether these learning differences cause language disorders, or whether they are a consequence of the same (see Outstanding Questions).

Corticostriatal dysfunctions have also been noted in psychiatric and other neurodevelopmental disorders, such as schizophrenia, obsessive-compulsive disorders, Tourette's disorder, and attention-deficit/hyperactivity disorder (ADHD) [90]. However, different computational models explain the behavioural learning profile in each of these disorders. For instance, dysfunctions of the ventral striatum and orbitofrontal/prefrontal cortices are linked to ADHD, but Tourette's disorder is better explained by an imbalance of the direct/indirect pathways [90]. It is not yet clear what distinct corticostriatal circuit dysfunction might distinguish language disorders from these other disorders with that exhibit very different symptomatology. One way to probe the specificity of learning impairments in developmental language disorders is to use learning tasks that are known to pattern with specific brain regions or pathways. Our working hypothesis is that developmental language disorders are more likely to be associated with corticostriatal loops involving the dorsal striatum, and that learning impairments in this group will be more evident when stimulus–response associations rather than state values must be learned.

Probing learning in these groups is likely to be helpful for designing better intervention. Different strategies are likely to be of benefit to typically developing children and those with language disorder. For example, studies with typically developing children suggest that greater variability in sentence structure is beneficial for learning syntax, but this variability did not aid children with language disorders 2, 11. Comprehending the nature of learning difficulties in children with language disorders will allow us to design interventions to circumvent these issues. Understanding the neurobiological interactions between learning systems might also offer insight into what might be optimal strategies. Studies of patients with acquired striatal or MTL damage suggest that altering the way we present information in a task, for instance, by changing the timing [91] or valence of feedback [92], affects learning performance as the relative involvement of striatal and MTL systems is changed. We need fMRI studies on children with SLI and dyslexia that use tasks that tap into language learning, and that are known to activate striatal or MTL systems. These will be key to understanding whether and how these learning strategies might alter learning outcomes for those with language disorders.

Outstanding Questions Are procedural learning difficulties a cause of language learning difficulties? The alternative explanations are that they co-occur with developmental language disorders, or are a consequence of language disorders.

Are procedural learning difficulties specific to language disorders? Procedural learning impairments have been reported in a range of different neurodevelopmental disorders such as autism, ADHD, and Williams syndrome. Is procedural learning particularly vulnerable during development? If this is the case, is there a set of procedural learning difficulties that distinguish language disorders from other neurodevelopmental disorders?

On a related note, what corticostriatal dysfunctions are specific to developmental language disorders? Corticostriatal dysfunctions are observed in psychiatric and other neurological disorders, for example Tourette's disorder, addiction, and Parkinson's disease. What is the best network model to explain the behavioural difficulties faced by children with developmental language disorders?

Are abnormalities in the structure and function of corticostriatal systems linked to individual differences in learning? What behavioural measures and brain activities are reliable indices of procedural and declarative learning systems?

Are there interactions between learning systems that can be exploited for learning? If so, why do relatively typical hippocampal learning systems not compensate adequately in developmental language disorders?

Can the conditions that promote learning in neurotypical individuals be applied to aid those with developmental language disorders, or do the conditions that benefit learning differ?

When learning sequential and non-sequential information, do children with language disorders engage different neurobiological learning systems? What makes one system resilient and not the other?

## Box 1 Why Use Developmental Dyslexia and SLI as Examples of Language Learning Disorders?

SLI is diagnosed when oral language lags behind other areas of development for no apparent reason, and is not the result of known developmental concerns, sensory impairments, or global developmental delay. SLI affects between 3–10% of children entering school [93]. Developmental dyslexia is identified when a child has poor literacy skills despite adequate intelligence and opportunity to learn, and similar prevalence levels are reported for this group [94]. Although the reading difficulties in dyslexia were initially thought to be in the visual perception domain, it is now fairly well established that most children with dyslexia have problems in the linguistic domain, particularly for categorising sounds, establishing sound–letter mappings, and manipulating sound sequences in their first language. These definitions indicate that both groups have specific difficulties with aspects of language learning. In addition, both disorders have a strong genetic basis, particularly for cases where nonword repetition deficits are noted [94].

There is a great deal of overlap between dyslexia and SLI. At a cognitive level, both groups show core deficits in phonology, particularly on tasks that tap into the ability to understand the sound structure of words (phonological awareness) and those that involve nonword repetition (motor control and phonological memory). Oral language problems place children at risk for literacy problems, both in reading comprehension and accuracy [95]. On the other hand, children at risk of dyslexia often experience early language difficulties, even before they start to read [96]. However, these disorders are not identical or even points on a continuum of phonological ability. Bishop and Snowling [94] have argued that these disorders are best characterised using a 2D model of phonological and non-phonological language skills. While children with dyslexia have typical non-phonological skills but impaired phonological ability, children with SLI are more likely to present with impairments of both phonological and non-phonological skills (for example, semantic and syntactic difficulties). Ramus and colleagues [74] have provided empirical support for this theory, but posit further dimensions with respect to phonological skills, separating out phonological representations and access.

Language problems are of significant concern in both SLI and dyslexia because poor language learning has been linked to negative consequences for academic achievement, self-esteem, social and emotional development, and employment [97]. Understanding why language learning is affected, and the specific mechanisms that impair their learning, could allow us to design optimal means of compensating for these difficulties.

## Box 2 Procedural/Declarative Distinctions in Memory

Initial distinctions between learning systems were based on studies of a noted patient, Henry Molaison, who became densely amnesic after bilateral hippocampectomy [98]. While he could not acquire new information such as facts and events, he was able to learn new visuomotor skills in rotary pursuit, bimanual tracking, and tapping tasks [99]. This led to a distinction being made between memory systems – ones that encoded explicit knowledge about facts and events, termed ‘declarative’ learning systems, and ‘procedural’ systems, which encoded skill-based information that is implicitly expressed through performance [100].

Ever since, the acquisition of declarative memory has been shown to depend upon heavily interconnected MTL structures, including the hippocampus, entorhinal, perirhinal, and parahippocampal cortices [101]. These regions are well adapted for rapid learning, and are particularly relevant when learning occurs over a single exposure, when associations between stimuli must be made over space or time, when acquired information must be used flexibly, and when learning depends on the awareness of what is being learned (reviewed in [102]). An umbrella term of ‘non-declarative’ learning is used to describe learning that can be accomplished without the MTL system, and this includes procedural learning of skills and habits, perceptual learning and priming, and classical conditioning [102]. Procedural learning is specifically used to describe a gradual trial-and-error learning system that leads to the formation of habits, which uses the reward related circuitry through the striatum. Knowledge acquired through this system is rigid and not verbalisable [102].

In some situations, both ‘declarative’ and ‘procedural’ systems can achieve learning, for example, tasks learned via memorisation can also be learned using a gradual trial-and-error strategy. The nature of the interactions between these two systems differ based on the information to be learned and the stage of learning [103]. Making optimal transitions between procedural and declarative memory could explain improvements in performance. When solving arithmetic problems, children must switch from a procedure-based counting strategy to a memory-based problem-solving one. In an imaging study [104], increased hippocampal–neocortical connectivity was longitudinally associated with a switch to memory-based strategies. With respect to language, more-efficient proceduralisation could accelerate language learning because speech actions could be chunked into sequences earlier in time. While there is no direct evidence for this, a recent study offers some indirect insight. Humans with a mutation of the *FOXP2* gene present with speech/language disorders and have difficulties learning and performing sequences of orofacial movements [105]. Mice carrying the humanised version of FOXP2 could more readily use procedural strategies in a cue-enriched T-maze task than could wild mice [106].

## Box 3 The Basal Ganglia and Their Role in Learning

The basal ganglia are a group of highly interconnected subcortical nuclei, including the striatum (caudate nucleus, putamen, and nucleus accumbens), globus pallidus, substantia niagra, and subthalamic nucleus. The basal ganglia interact with the cortex in parallel processing loops where regions of the cortex project to the striatum, which in turn project to the pallidum and then to the thalamus, and then on to frontal cortex. Such loops through the basal ganglia have traditionally been associated with motor control, particularly the selection and suppression of actions. However, the function of these loops is complementary to the cortical areas that project to the loop. The ventral striatum (nucleus accumbens) receives input from the orbitofrontal cortex, temporal cortex, amygdala, and hippocampus, and is thought to gate behaviour in response to emotional or motivational stimuli. Particularly relevant for learning are loops through the basal ganglia that take on sensorimotor, executive or associative, and limbic functions.

The basal ganglia have been implicated in learning gained through practice, such as the formation of skill memories or habits. Lesion studies in rodents indicate that the basal ganglia are necessary for habit formation [107]. In humans, both neuroimaging and neuropsychological studies demonstrate that the basal ganglia play a key role in learning sequences of actions or stimulus–response mappings. Initially, tasks used to probe learning were implicit, which led to the view that the basal ganglia was specialised for non-declarative learning. However, this did not reconcile with findings where the basal ganglia were recruited in explicit sequence learning or categorisation tasks. The findings highlighting the role of the striatum in learning stimulus–response mapping through reinforcement [108] have helped to reconcile these differences [109]. Recently, Graybiel and colleagues [110] have proposed that the striatum could play a role in optimising behaviour over time by implementing reinforcement-based feedback to allow an effective combination of sequential motor elements. Subdivisions of the striatum are thought to contribute differently to learning. While the ventral striatum contributes to the initial learning of a motivated behaviour, the dorsal striatum plays a role in initial learning from anticipated outcome of behaviours for future selection, and later in creating a habitual pattern that is resistant to interference.

## Box 4 Subcomponents of Language Learning

*Learning Speech Sounds*

Language users learn to divide up acoustic information into sound categories that are relevant in the language. A phoneme is the smallest unit that changes word meaning in a language. Phonemes are not universal across languages, for example, /r/-/l/ are not distinguished in Japanese, and native Japanese speakers group them together in a single category. However, these sounds distinguish words in English. Listeners must use a combination of acoustic features to map specific sounds onto a speech category. Listeners must also learn to produce these sounds within the constraints of the speech category boundaries of their language.

*Learning Phonology*

Phonemes are sequenced together to form words, but these combinations follow language-specific constraints. For instance, /mb/ is not a legal combination to start a word with in English. Language learners will implicitly extract these constraints, which are called the phonotactics of a language. Learning these regularities helps word learning. These regularities are also important for production because it is easier to produce words that have higher phonotactic probability.

*Learning Vocabulary*

Word learning involves making an arbitrary connection between a sequence of sounds and a concept, which may be concrete or abstract. Mappings are usually not explicitly taught, but are learned implicitly in complex environments, and listeners must therefore track regularities between the occurrence of a word and its referent. In addition, words are typically extracted from a continuous stream of speech, and listeners use multiple cues – transitions between phonemes, grammatical cues, prosodic boundaries – to discern word boundaries. Once an association between a word and its referent is learned, language users must also be able to retrieve it in expressive speech and writing.

*Learning Syntax*

To interpret and use a language, listeners must build an understanding of sentence structure. While initial use of syntax may only comprise a set of exemplar sentence frames; with time and increased language experience, learners will detect statistical regularities in their input and use these to construct more abstract patterns or structures. A key challenge in learning grammar is that it is not merely patterns between adjacent words that must be learned, but also patterns that are non-adjacent and occur over intervening words. Again, language users must be able to use these structures in speech or writing.

*Learning Pragmatics*

Language is primarily a social tool, and our use of language changes based on whether we are speaking or writing, with whom we are communicating, and the context in which we are speaking. Language learners must learn which constructions are appropriate to produce in different social and functional settings, and must learn to use context to infer what is not directly stated.

## Glossary

Artificial grammar learning (AGL): implicit learning task where a set of rules specifies how different elements in a sequence can combine. After being exposed to a training set comprising legal combinations of sequences (rules are not explicitly revealed), participants are asked to judge the grammaticality of novel sequences. If participants have acquired tacit knowledge, their performance will be higher than chance.
Contextual cueing: participants search for a visual target in a complex visual display. Across trials, some spatial configurations of distractor objects are repeated; targets appear in consistent locations within these arrays. Targets appearing in repeated configurations are detected faster as participants learn associations between spatial configurations and target locations. Learning is implicit because the configurations are not explicitly recognisable.
Eyeblink conditioning: Pavlovian classical conditioning paradigm in which a once-neutral stimulus comes to elicit a learned reflexive response. Typically, an individual is presented with a conditioned stimulus (a tone) followed by the unconditioned stimulus, a puff of air, which elicits a reflexive eyeblink. After several pairings, a conditioned eyeblink response will be evoked by the conditioned stimulus, even before the presentation of the airpuff.
Hebb repetition learning: this is a well-known sequence repetition paradigm that is thought to share the mechanisms underlying word learning. The paradigm examines how participants retain stable chunks of information over time. Sequences of words, digits, or letters are administered for immediate recall, with some sequences being repeated. Participants typically remain unaware of the repetition but demonstrate implicit learning by better recall of repeated than non-repeated sequences.
Paired associate learning: declarative learning paradigm requiring the pairing of a stimulus and response item in memory. Pairs can be in the same modality or crossmodal (e.g., visual–verbal).
Probabilistic categorical learning tasks: participants learn to map multi-feature stimuli to an outcome. Because mappings are probabilistic, learning one-to-one mappings does not lead to efficient learning. Instead, trial-by-trial feedback must be used to gradually learn the associations between combinations of features of the stimuli and their outcomes.
Pursuit rotor task: visuomotor procedural learning task where participants must track a moving target on a turntable with a stylus (or an on-screen target with a cursor). The target moves in a circular path, and participants learn to appropriately adjust hand movements according to upcoming visual information. The dependent measure is the duration participants make contact with the target.
Serial reaction time (SRT): assessments of visuomotor sequence procedural learning. Participants respond by pressing a button that spatially corresponds to a visual stimulus which can appear in one of four locations. In some blocks, sequences of visual signals are repeated, which leads to a reduction in reaction time on button presses. Participants typically are unaware of the repeated sequence. In a subsequent block, random sequences are reintroduced, and reaction time rebounds.
Statistical learning: in this form of learning, structured regularities about the frequency and co-occurrence of different exemplars are extracted from the input, even when participants have no intention to learn. Such learning is automatic, spontaneous, can occur merely through observation, and participants are typically unaware that they have extracted any structure. In the statistical learning tasks described here, the regularities to be extracted are high transitional probabilities between elements in a sequence, whereas low transitional probabilities indicate ‘word’ boundaries. Participants judge the familiarity of ‘word’ and ‘part-word’ chunks.

## References

[1] Kan P.F., Windsor J. (2010). Word learning in children with primary language impairment: a meta-analysis. J. Speech Lang. Hear. Res..

[2] Hsu H.J., Bishop D.V. (2014). Training understanding of reversible sentences: a study comparing language-impaired children with age-matched and grammar-matched controls. Peer J..

[3] Ullman M.T., Pierpont E.I. (2005). Specific language impairment is not specific to language: the procedural deficit hypothesis. Cortex.

[4] Pinker S., Ullman M.T. (2002). The past and future of the past tense. Trends Cogn. Sci..

[5] Lum J.A.G. (2014). Procedural learning deficits in specific language impairment (SLI): a meta-analysis of serial reaction time task performance. Cortex.

[6] Gabriel A. (2013). Procedural learning in specific language impairment: effects of sequence complexity. J. Int. Neuropsychol. Soc..

[7] Hsu H.J., Bishop D.V. (2014). Sequence-specific procedural learning deficits in children with specific language impairment. Dev. Sci..

[8] Nicolson R.I., Fawcett A.J. (2007). Procedural learning difficulties: reuniting the developmental disorders?. Trends Neurosci..

[9] Lum J.A.G. (2013). Procedural learning is impaired in dyslexia: evidence from a meta-analysis of serial reaction time studies. Res. Dev. Disabil..

[10] Pavlidou E.V. (2010). Do children with developmental dyslexia have impairments in implicit learning?. Dyslexia.

[11] Grunow H. (2006). The effects of variation on learning word order rules by adults with and without language-based learning disabilities. J. Commun. Disord..

[12] Plante E. (2002). Sensitivity to word order cues by normal and language/learning disabled adults. J. Commun. Disord..

[13] Pavlidou E.V., Williams J.M. (2014). Implicit learning and reading: insights from typical children and children with developmental dyslexia using the artificial grammar learning (AGL) paradigm. Res. Dev. Disabil..

[14] Hsu H.J. (2014). Impaired statistical learning of non-adjacent dependencies in adolescents with specific language impairment. Front. Psychol..

[15] Evans J. (2009). Statistical learning in children with specific language impairment. J. Speech Lang. Hear. Res..

[16] Gabay Y. (2015). Impaired statistical learning in developmental dyslexia. J. Speech Lang. Hear. Res..

[17] Desmottes L. (2016). Later learning stages in procedural memory are impaired in children with specific language impairment. Res. Dev. Disabil..

[18] Hedenius M. (2011). Grammar predicts procedural learning and consolidation deficits in children with specific language impairment. Res. Dev. Disabil..

[19] Hedenius M. (2013). Impaired implicit sequence learning in children with developmental dyslexia. Res. Dev. Disabil..

[20] Tomblin J.B. (2007). Procedural learning in adolescents with and without specific language impairment. Lang. Learn. Dev..

[21] Conti Ramsden G. (2015). The relation between receptive grammar and procedural, declarative, and working memory in specific language impairment. Front. Psychol..

[22] Lum J.A.G. (2012). Working, declarative and procedural memory in specific language impairment. Cortex.

[23] Mainela-Arnold E., Evans J. (2013). Do statistical segmentation abilities predict lexical-phonological and lexical-semantic abilities in children with and without SLI?. J. Child Lang..

[24] Ullman M.T., Pullman M.Y. (2015). A compensatory role for declarative memory in neurodevelopmental disorders. Neurosci. Biobehav. Rev..

[25] Lum J.A.G. (2015). Verbal declarative memory impairments in specific language impairment are related to working memory deficits. Brain Lang..

[26] Bishop D.V.M., Hsu H.J. (2015). The declarative system in children with specific language impairment: a comparison of meaningful and meaningless auditory-visual paired associate learning. BMC Psychol..

[27] Litt R.A., Nation K. (2014). The nature and specificity of paired associate learning deficits in children with dyslexia. J. Memory Lang..

[28] Kahta S., Schiff R. (2016). Implicit learning deficits among adults with developmental dyslexia. Ann. Dyslexia.

[29] Jiménez-Fernández G. (2010). Dyslexic children show deficits in implicit sequence learning, but not in explicit sequence learning or contextual cueing. Ann. Dyslexia.

[30] Howard J.H. (2006). Dyslexics are impaired on implicit higher-order sequence learning, but not on implicit spatial context learning. Neuropsychologia.

[31] Nigro L. (2016). Implicit learning of non-linguistic and linguistic regularities in children with dyslexia. Ann. Dyslexia.

[32] Lee J.C., Tomblin J.B. (2015). Procedural learning and individual differences in language. Lang. Learn. Dev..

[33] Hardiman M.J. (2013). Children with specific language impairment are not impaired in the acquisition and retention of Pavlovian delay and trace conditioning of the eyeblink response. Brain Lang..

[34] Steinmetz A.B., Rice M.L. (2010). Cerebellar-dependent delay eyeblink conditioning in adolescents with specific language impairment. J. Neurodev. Disord..

[35] Gabay Y., Holt L.L. (2015). Incidental learning of sound categories is impaired in developmental dyslexia. Cortex.

[36] Gabay Y. (2015). Probabilistic category learning in developmental dyslexia: evidence from feedback and paired-associate weather prediction tasks. Neuropsychology.

[37] Lee J.C., Tomblin J.B. (2012). Reinforcement learning in young adults with developmental language impairment. Brain Lang..

[38] Mayor-Dubois C. (2014). Nondeclarative learning in children with specific language impairment: predicting regularities in the visuomotor, phonological, and cognitive domains. Child Neuropsychol..

[39] Packard M.G., Knowlton B.J. (2002). Learning and memory functions of the basal ganglia. Annu. Rev. Neurosci..

[40] Cunillera T. (2009). Time course and functional neuroanatomy of speech segmentation in adults. NeuroImage.

[41] McNealy K. (2006). Cracking the language code: neural mechanisms underlying speech parsing. J. Neurosci..

[42] Karuza E.A. (2013). The neural correlates of statistical learning in a word segmentation task: an fMRI study. Brain Lang..

[43] De Diego-Balaguer R. (2008). Striatal degeneration impairs language learning: evidence from Huntington's disease. Brain.

[44] Kalm K. (2013). Individual sequence representations in the medial temporal lobe. J. Cogn. Neurosci..

[45] Rauschecker J.P., Scott S.K. (2009). Maps and streams in the auditory cortex: nonhuman primates illuminate human speech processing. Nat. Neurosci..

[46] López-Barroso D. (2015). Multiple brain networks underpinning word learning from fluent speech revealed by independent component analysis. NeuroImage.

[47] Plante E. (2014). Dynamic changes in network activations characterize early learning of a natural language. Neuropsychologia.

[48] Opitz B., Friederici A.D. (2003). Interactions of the hippocampal system and the prefrontal cortex in learning language-like rules. NeuroImage.

[49] Breitenstein C. (2005). Hippocampus activity differentiates good from poor learners of a novel lexicon. NeuroImage.

[50] Wing E.A. (2013). Neural correlates of retrieval-based memory enhancement: an fMRI study of the testing effect. Neuropsychologia.

[51] Davis M.H. (2009). Learning and consolidation of novel spoken words. J. Cogn. Neurosci..

[52] Davis M.H., Gareth Gaskell M. (2009). A complementary systems account of word learning: neural and behavioural evidence. Philos. Trans. R. Soc. Lond. B Biol. Sci..

[53] O’Kane G. (2004). Evidence for semantic learning in profound amnesia: an investigation with patient H.M. Hippocampus.

[54] Vargha-Khadem F. (1997). Differential effects of early hippocampal pathology on episodic and semantic memory. Science.

[55] Ripollés P. (2014). The role of reward in word learning and its implications for language acquisition. Curr. Biol..

[56] Tricomi E., Fiez J.A. (2011). Information content and reward processing in the human striatum during performance of a declarative memory task. Cogn. Affect. Behav. Neurosci..

[57] Tricomi E., Fiez J.A. (2008). Feedback signals in the caudate reflect goal achievement on a declarative memory task. NeuroImage.

[58] Rauschecker A.M. (2008). Changes in neural activity associated with learning to articulate novel auditory pseudowords by covert repetition. Hum. Brain Mapp..

[59] Simmonds A.J. (2014). The response of the anterior striatum during adult human vocal learning. J. Neurophysiol..

[60] Yi H-G. (2016). The role of corticostriatal systems in speech category learning. Cereb. Cortex.

[61] Tricomi E. (2006). Performance feedback drives caudate activation in a phonological learning task. J. Cogn. Neurosci..

[62] Mayes A.K. (2015). Neural correlates of childhood language disorder: a systematic review. Dev. Med. Child Neurol..

[63] Richlan F. (2011). Meta-analyzing brain dysfunctions in dyslexic children and adults. NeuroImage.

[64] Jernigan T.L. (1991). Cerebral structure on magnetic resonance imaging in language- and learning-impaired children. Arch Neurol..

[65] Badcock N.A. (2012). Co-localisation of abnormal brain structure and function in specific language impairment. Brain Lang..

[66] Soriano-Mas C. (2009). Age-related brain structural alterations in children with specific language impairment. Hum. Brain Mapp..

[67] Lee J.C. (2013). Abnormal subcortical components of the corticostriatal system in young adults with DLI: a combined structural MRI and DTI study. Neuropsychologia.

[68] Watkins K.E. (2002). MRI analysis of an inherited speech and language disorder: structural brain abnormalities. Brain.

[69] Northam G.B. (2012). Interhemispheric temporal lobe connectivity predicts language impairment in adolescents born preterm. Brain.

[70] Richlan F. (2012). Structural abnormalities in the dyslexic brain: a meta-analysis of voxel-based morphometry studies. Hum. Brain Mapp..

[71] Jednoróg K. (2015). How reliable are gray matter disruptions in specific reading disability across multiple countries and languages? Insights from a large-scale voxel-based morphometry study. Hum. Brain Mapp..

[72] Hebb A.O., Ojemann G.A. (2013). The thalamus and language revisited. Brain Lang..

[73] Tamboer P. (2015). Dyslexia and voxel-based morphometry: correlations between five behavioural measures of dyslexia and gray and white matter volumes. Ann. Dyslexia.

[74] Ramus F. (2013). Phonological deficits in specific language impairment and developmental dyslexia: towards a multidimensional model. Brain.

[75] Nation K. (2010). A longitudinal investigation of early reading and language skills in children with poor reading comprehension. J. Child Psychol. Psychiatry.

[76] Thompson P.A. (2015). Developmental dyslexia: predicting individual risk. J. Child Psychol. Psychiatry.

[77] Kita Y. (2013). Altered brain activity for phonological manipulation in dyslexic Japanese children. Brain.

[78] de Guibert C. (2011). Abnormal functional lateralization and activity of language brain areas in typical specific language impairment (developmental dysphasia). Brain.

[79] Dibbets P. (2006). Functional MRI of task switching in children with specific language impairment (SLI). Neurocase.

[80] Hosseini S.M.H. (2013). Topological properties of large-scale structural brain networks in children with familial risk for reading difficulties. NeuroImage.

[81] Krafnick A.J. (2011). Gray matter volume changes following reading intervention in dyslexic children. NeuroImage.

[82] Gebauer D. (2012). Differences in brain function and changes with intervention in children with poor spelling and reading abilities. PLoS ONE.

[83] Gauger L.M. (1997). Brain morphology in children with specific language impairment. J. Speech Lang. Hear. Res..

[84] Hugdahl K. (2004). fMRI brain activation in a Finnish family with specific language impairment compared with a normal control group. J. Speech Lang. Hear. Res..

[85] Girbau-Massana D. (2014). Gray–white matter and cerebrospinal fluid volume differences in children with specific language impairment and/or reading disability. Neuropsychologia.

[86] Ellis Weismer S. (2005). A functional magnetic resonance imaging investigation of verbal working memory in adolescents with specific language impairment. J. Speech Lang. Hear. Res..

[87] Verhoeven J.S. (2012). Is there a common neuroanatomical substrate of language deficit between autism spectrum disorder and specific language impairment?. Cereb. Cortex.

[88] Vydrova R. (2015). Structural alterations of the language connectome in children with specific language impairment. Brain Lang..

[89] Dubois J., Adolphs R. (2016). Building a science of individual differences from fMRI. Trends Cogn. Sci..

[90] Maia T.V., Frank M.J. (2011). From reinforcement learning models to psychiatric and neurological disorders. Nat. Neurosci..

[91] Foerde K. (2013). A role for the medial temporal lobe in feedback-driven learning: evidence from amnesia. J. Neurosci..

[92] Frank M.J. (2004). By carrot or by stick: cognitive reinforcement learning in parkinsonism. Science.

[93] Tomblin J.B. (1997). Prevalence of specific language impairment in kindergarten children. J. Speech Lang. Hear. Res..

[94] Bishop D.V., Snowling M.J. (2004). Developmental dyslexia and specific language impairment: same or different?. Psychol. Bull..

[95] Catts H.W. (1993). The relationship between speech-language impairments and reading disabilities. J. Speech Lang. Hear. Res..

[96] Nash H.M. (2013). Preschool language profiles of children at family risk of dyslexia: continuities with specific language impairment. J. Child Psychol. Psychiatry.

[97] Conti Ramsden G. (2013). Adolescents with a history of specific language impairment (SLI): strengths and difficulties in social, emotional and behavioral functioning. Res. Dev. Disabil..

[98] Scoville W.B., Milner B. (1957). Loss of recent memory after bilateral hippocampal lesions. J. Neurol. Neurosurg. Psychiatry.

[99] Corkin S. (1968). Acquisition of motor skill after bilateral medial temporal-lobe excision. Neuropsychologia.

[100] Corkin S. (2002). What's new with the amnesic patient H.M?. Nat. Rev. Neurosci..

[101] Squire L.R., Zola-Morgan S. (1991). The medial temporal lobe memory system. Science.

[102] Squire L.R., Dede A.J.O. (2015). Conscious and unconscious memory systems. Cold Spring Harb. Perspect. Biol..

[103] Shohamy D. (2008). Basal ganglia and dopamine contributions to probabilistic category learning. Neurosci. Biobehav. Rev..

[104] Qin S. (2014). Hippocampal–neocortical functional reorganization underlies children's cognitive development. Nat. Neurosci..

[105] Watkins K.E. (2002). Behavioural analysis of an inherited speech and language disorder: comparison with acquired aphasia. Brain.

[106] Schreiweis C. (2014). Humanized Foxp2 accelerates learning by enhancing transitions from declarative to procedural performance. Proc. Natl. Acad. Sci..

[107] Yin H.H., Knowlton B.J. (2006). The role of the basal ganglia in habit formation. Nat. Rev. Neurosci..

[108] Schultz W. (1997). A neural substrate of prediction and reward. Sci. New Series.

[109] Shohamy D. (2011). Learning and motivation in the human striatum. Curr. Opin. Neurobiol..

[110] Graybiel A.M., Grafton S.T. (2015). The striatum: where skills and habits meet. Cold Spring Harb. Perspect. Biol..

